# The Pathology and Surgical Treatment of Tumors

**Published:** 1896-02

**Authors:** 


THE PATHOLOGY AND SURGICAL TREATMENT OF TUMORS. BY N. SENN, M. D., Ph. D., LL. D., Professor of Practice of Surgery and Clinical Surgery, Rush Medical College ; Professor of Surgery, Chicago Polyclinic ; Attending Surgeon to Presbyterian Hospital ; Surgeon-in-Chief, St. Joseph's Hospital, Chicago. Octavo, pp. 709. Illustrated by 515 engravings, including full-page colored plates. Price, cloth, $6.00 ; half-morocco, $7.00. Philadelphia : W. B. Saunders, 925 Walnut street. 1895.

This is a volume of some 700 pages, with over 500 illustrations, 100 of which are new and original, while the remainder are carefully selected. Noticeable among them are many admirable reproductions of photographs and photomicrographs, giving with diagramatic drawings a very satisfactory picture of the histological and gross appearances of the various forms of tumors. The work is planned to be a text-book for students as well as a reference book for physicians and surgeons to aid in diagnosis and guide in operations. Some of the more important and difficult operations are fully described and illustrated.

The first section is purely didactic, taking up the anatomy, biology, etiology and pathology of tumors in general with the various methods of treatment. Then follows a chapter on classification, concluding with the author's very simple and sensible arrangement of all tumors into practically four groups: first, those of epiblastic and hypoblastic origin ; second, those of mesoblastic origin ; third, the teratomata ; and fourth, retention cysts. The latter, however, he does not consider as true tumors.

The individual forms are then taken up separately with diagnosis, prognosis and treatment, thus covering the ground very thoroughly and satisfactorily.

One of the features of the work which makes it especially valuable to the student and general practitioner is the fact that much space is given to the consideration of the clinical distinction between true tumors, inflammatory swellings and retention cysts ; as also to the topographical distribution of the various kinds of tumors in the different regions and organs of the body.

Altogether, it is a commendable effort to combine in convenient form information that otherwise would have to be sought for in treatises on pathology and surgery at considerable expense of time, and the author is to be congratulated upon the successful way in which he has accomplished his work. H. A. M.

